# F80. COGNITIVE TRAJECTORIES OVER 6 YEARS IN FIRST-EPISODE SCHIZOPHRENIA AND HEALTHY CONTROLS – A PROSPECTIVE LONGITUDINAL MULTI-ASSESSMENT STUDY

**DOI:** 10.1093/schbul/sby017.611

**Published:** 2018-04-01

**Authors:** Susie Fu, Nikolai Czajkowski, Anne-Kari Torgalsboen

**Affiliations:** University of Oslo

## Abstract

**Background:**

Patients with first-episode schizophrenia (FES) have consistently showed impaired cognitive functioning compared to healthy controls across a broad array of cognitive domains. After psychosis onset the cognitive performance in FES seems to remain stable or even improve over time. Many earlier studies, however, did not include healthy control groups which made it unclear whether cognitive changes were due to genuine improvements or other arbitrary factors. Thus, the development of individual cognitive domains over time is not yet fully examined.

**Methods:**

The present study has a multi-assessment design, and includes data from eight follow-ups over six years. For the patient group, assessments were conducted yearly, apart from the first year where assessments were conducted every six months. Healthy controls were assessed at baseline, after two years and after six years. A total of 28 FES-patients and 28 healthy controls participated in the study, with 79 % of patients retained at the 6-year follow-up. Cognition was assessed with MATRICS Consensus Cognitive Battery. Data were analyzed with linear multilevel models.

**Results:**

FES-patients scored lower than the control group across all cognitive domains at baseline. Over six years, the cognitive trajectories of visual learning seem to remain stable for both groups, while FES-patients showed slight improvements in attention (β = 1.34, SE = .18, p < .001), verbal learning (β = .65, SE = .29, p < .031), processing speed (β = .69, SE = .35, p < .051), reasoning/ problem solving (β = 1.68, SE = .27 p < .001), working memory (β = .89, SE = .27, p < .002) and social cognition (β = .93, SE = .30, p < .003). Most of these cognitive trajectories start to improve within the first year of illness and continues throughout the six year period. The improvement in processing speed (β = .18, SE = .48, p > .05), verbal learning (β = .56, SE = .59, p > .05) and social cognition (β = .82, SE = .59, p > .05) seem to be larger for FES-patients compared to controls, but these differences were not significant. The patient group’s improvement in reasoning/ problem solving (β = 1.31, SE = .51, p < .05) was significantly larger that the control group, but they showed smaller improvement in working memory (β = -1.03, SE = .51, p < .05).

**Discussion:**

Our results show that improvements are already discernable after 6 months following illness outbreak. There are different trajectories for different cognitive domains. Moreover, two cognitive domain trajectories were significantly different between control group and FES-patients. This points to the importance of assessing cognitive development over many years with multiple assessments when exploring cognitive impairments in schizophrenia. From a clinical perspective, this may speak in favor of a targeted rehabilitation of different cognitive domains.

